# Embossed Mie resonator arrays composed of compacted TiO_2_ nanoparticles for broadband anti-reflection in solar cells

**DOI:** 10.1038/s41598-020-69518-6

**Published:** 2020-07-27

**Authors:** Dennis Visser, Ding Yuan Chen, Yohan Désières, Ajith Padyana Ravishankar, Srinivasan Anand

**Affiliations:** 1 Department of Applied Physics, KTH Royal Institute of Technology, Electrum 229, 164 40 Kista, Sweden; 2 University Grenoble Alpes, CEA, LETI, MINATEC Campus, 38054 Grenoble, France

**Keywords:** Optical materials and structures, Nanoscale materials, Semiconductors, Surface patterning, Solar energy and photovoltaic technology

## Abstract

Mie resonator arrays formed by embossing titanium dioxide (TiO_2_) nanoparticles (NPs) from solution are investigated as optical coatings for anti-reflection applications. Compacted nanoparticle assemblies offer unique possibilities to tailor the effective refractive index (RI). Here, we demonstrate a simple table-top, low pressure, and low temperature method to fabricate structured optical coatings. TiO_2_ nanostructures in the form of nanodisks support Mie resonances in the visible wavelength spectrum and exhibit strong forward scattering into the high index substrates, making them suitable as broadband anti-reflection coatings for solar cells. TiO_2_ NP-based nanodisk arrays are designed, fabricated, and characterized regarding their anti-reflection properties on Si, GaAs, and InP substrates and solar cells. Detailed finite-difference time-domain simulations are performed to optimize the TiO_2_ NP-based Mie resonator arrays for the broadband anti-reflection as well as to explain the measured reflectance spectra. The solar-weighted reflectance is used as a figure of merit (FoM). TiO_2_ nanodisk arrays on Si show a FoM of ~ 7% in the 400–1,100 nm wavelength spectrum; similar values are obtained for GaAs and InP substrates. TiO_2_ nanodisk arrays embossed directly on prefabricated planar single-junction Si, GaAs, and InP solar cells result in an appreciable increase (~ 1.3 times) in the short-circuit current densities.

## Introduction

Recently, sub-wavelength dielectric Mie resonator arrays have been reported for applications such as omnidirectional broadband anti-reflection.^1,2^ Si nanodisk arrays on Si substrates show low average surface reflectance over the visible-NIR wavelength region.^3^ Surface reflection reduction^4-7^ plays a major part in increasing the performance of solar cells. For inorganic semiconductor solar cell materials, e.g., Si and III-Vs, due to their high refractive indices (~ 3–4) the reflectance loss (~ 30–40%) is significant. To reduce this, the solar cell surface can either be structured directly or an additional (structured) optical coating can be used. Direct structuring of solar cells can degrade its performance due to process induced defects and surface recombination and invariably requires additional passivation procedures/coatings. Such issues become more significant for thin film solar cells (thickness of ~ 2 µm or less). These limitations may be overcome by depositing a structured optical layer instead. Anti-reflection coatings (ARCs) include commonly used traditional thin-film dielectrics (e.g., silicon dioxide (SiO_2_) and silicon nitride (Si_x_N_y_)), metal nanoparticles,^8,9^ and dielectric nanostructures^3,10,11^. While thin film dielectrics are easier to fabricate, multilayers are often required to achieve broadband anti-reflection. Metallic nanoparticles suffer from parasitic absorption in the metal and the resonances are highly sensitive to the RI of the matrix below or around the nanoparticles.^12^ High-index dielectric (e.g., Si) Mie resonator arrays placed on Si or substrates with similar refractive indices also absorb above bandgap light and thus have to be integrated in the solar cell.^3,11^ Thus, resonators made from materials which are non-absorbing in the useful part of the solar spectrum would be interesting. Dielectric nanoresonators with moderate (1.7 < n < 3.0) RIs can simultaneously support strong electric and magnetic resonances in the visible-NIR wavelength spectrum and forward scattering into the high index substrate.^1^ Amongst metal oxides, TiO_2_ is an interesting candidate due to its fairly high RI (n≈2.4–2.9) and transparency in the visible-NIR wavelength spectrum.^13-17^ TiO_2_ (nano)materials have been utilized in solar cell devices such as dye-sensitized solar cells, polymer-inorganic hybrid solar cells, quantum dot-sensitized solar cells, and perovskite solar cells,^13^ heterojunction solar cells with electron-selective TiO_2_ contact,^18^ photo electrochemical water splitting,^19^ as part of (graded-index) anti-reflection layer coating(s),^20-26^ quasi-periodic multilayer ARCs,^27^ light manipulation metasurfaces,^28-36^ and for light extraction in light emitting diodes (LEDs)^37-39^.

However, fabricating nanoresonators^40-44^ from TiO_2_ thin films would require deposition and subsequent patterning steps and may not be compatible with providing these structures on arbitrary substrates and prefabricated devices. On the other hand, soft imprinting or embossing methods offer a simple and elegant way to form Mie resonator arrays by compacting metal-oxide nanoparticles from solution.^39,41,43^ The effective RI of TiO_2_ NP films can be tuned by changing the type of polymorph (e.g., anatase or rutile), the (average) particle size, and/or fill factor (air-nanoparticle). By appropriate choice of molds, a variety of geometries – size, shape, and spacing (depending on the mold) – can be fabricated by soft-imprint/embossing. Furthermore, such embossed optical coatings can, in principle, be applied on different planar substrates including prefabricated devices. Additionally, if the embossing can be performed at low temperatures, problems due to mechanical stress and other thermally induced material degradation in the devices can be avoided.

In this work, microcone and nanodisk arrays composed of TiO_2_ NPs assemblies are investigated as anti-reflection coatings for Si and III-V (GaAs & InP) solar cells. A simple table-top, low pressure (~ 0.1 bar), and low temperature (< 100 °C) embossing method for fabricating these structures is demonstrated. The method reported here for fabricating ARCs, while having similar advantages of sol–gel methods, namely, cost-effectiveness and ease of fabrication,^21,24^ also enables three-dimensional (3D) structures to be formed. For broadband wide-angle anti-reflection applications, multilayer ARCs show a high sensitivity for their anti-reflection properties with respect to layer thickness variations.^45^ Nanostructured (e.g., Mie resonators) ARCs have the advantage of being less sensitive with regard to the angle of incidence of the incoming light. This makes them more useful for, e.g., solar cells used in combination with (large numerical aperture) optical concentrators. It has been previously shown that a design that integrates a nanostructured anti-reflection layer with a multilayer ARC results in an ultrabroadband and wide-angle ARC.^46^ The embossing technique reported in this work could be used for this type of design to simplify the fabrication of this last top 3D layer. Other methods for the structuring of TiO_2_ have been reported, though they show the disadvantage of high fabrication temperatures (> 300 °C), which may not be suitable for on-device fabrication, and/or is only demonstrated in combination with resin; thereby limiting the RI.^37,38^ Due to the low fabrication temperature used in this work, in principle, these type of optical coatings can be applied in a broader scope of material combinations than already reported. The optical characteristics of a spin coated TiO_2_ NPs-based layer on glass and Si are investigated with regard to its transmission and absorption properties, fill factor, and effective RI. The optical properties of the embossed structures on Si, GaAs, and InP substrates are investigated by spectrally-resolved reflectance measurements and finite-difference time-domain (FDTD) simulations. As a proof of principle, optical and electrical characterization of Si, GaAs, and InP solar cells embossed with the TiO_2_ NP-based optical coatings is included; where all three types of solar cells show an appreciable increase of the short-circuit current density (J_sc_).

### Optical properties of TiO_2_ NP thin films

**Figure 1:**
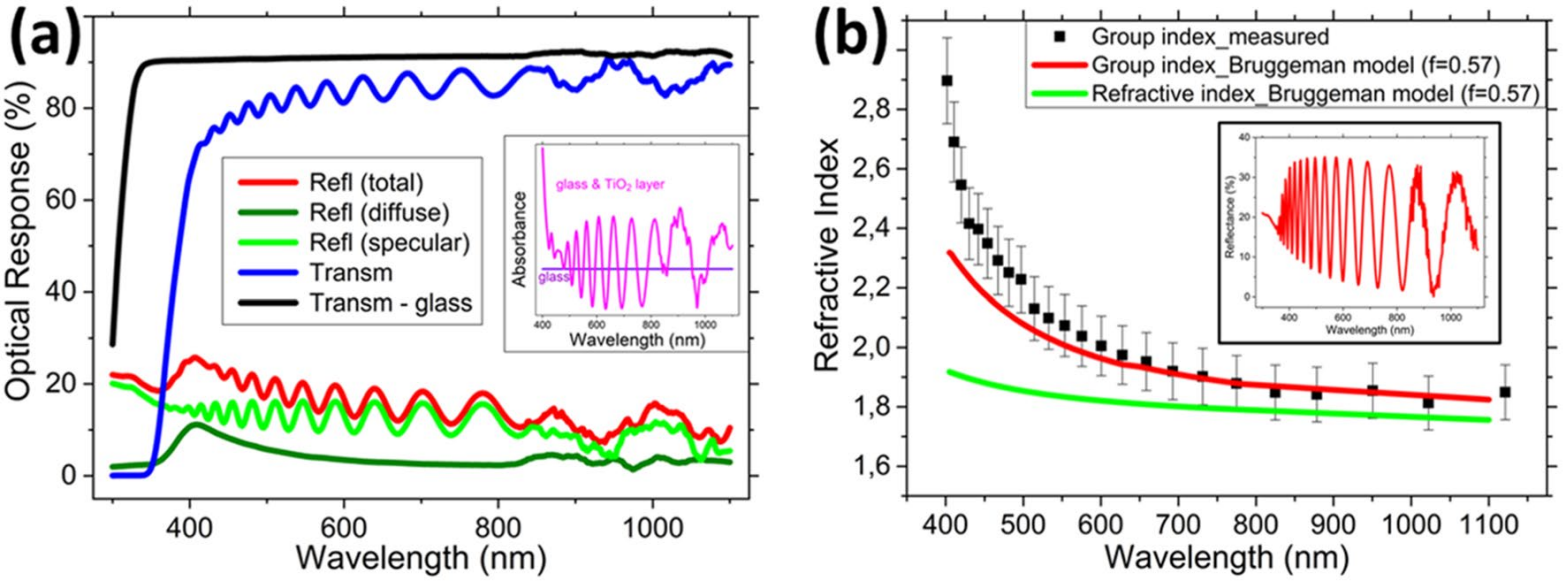
Optical characterization data of a spin coated composite mixture of anatase and rutile TiO_2_ NP-based layer assembly. (**a**) The reflectance (total (red), diffuse (dark green), and specular (light green)) and transmittance (blue) spectra for a TiO_2_ NP-based layer (~ 1.9 μm) spin coated on a glass substrate. The transmittance spectrum of glass (black) is included as a reference. Inset: the absorbance of the TiO_2_ NP-based layer (magenta) relative to glass (purple). (**b**) The determined RI of a TiO_2_ NP-based layer (~ 1.75 μm) spin coated on a Si substrate, obtained from the Fabry-Pérot oscillations (inset) and fitting based on a Bruggeman model.

The optical properties of TiO_2_ NP thin films (~ 1.9 μm thick) obtained by spin coating TiO_2_ NPs on a glass substrate, are investigated with regard to their reflectance, transmittance, and absorbance properties (Fig. 1a). Both total and specular reflectance spectra (at normal incidence) show clear Fabry-Pérot oscillations due to thin film interference and are related to the thickness and effective RI of the TiO_2_ NP thin film. For wavelengths < 550 nm an increased diffuse reflectance is observed and is attributed to Rayleigh backscattering; ~ 10% of scattering is obtained at a wavelength of 400 nm. The (total) transmittance of the TiO_2_ NP thin film indicates high transparency for wavelengths > 400 nm and a band-edge at ~ 380 nm. The absorbance spectra are obtained from the total reflectance and transmittance spectra and the absorbance relative to glass is shown in the inset of Fig. 1a, where the oscillations are due to interference effects. It can be concluded that the TiO_2_ NPs have negligible absorption for wavelengths > 400 nm.

Figure 1b shows the determined effective RI of a TiO_2_ NP film. The analysis was performed utilizing the reflectance spectra (see inset Fig. 1b) of a spin coated NP layer (~ 1.75 μm thick) on a Si substrate. A high index substrate (Si) was chosen since the Fabry-Pérot oscillations are more pronounced compared to that obtained on glass and the light transmitted into Si is fully absorbed in the visible wavelength spectrum. A scanning electron microscopy (SEM) image of the spin coated layer is included in Fig. S1 of the Supplementary Information. The group indices (and fill factor) were determined using the relative maxima/minima positions of the Fabry-Pérot oscillations and by fitting based on a Bruggeman model.^47,48^ The results in Fig. 1b indicate good agreement of the fitted data (red line) with the measured data (black markers) for wavelengths > 550 nm for a material fill factor of ~ 0.57 ± 0.05; which is somewhat below the maximum fill factor (0.64) for random packing of spherical nanoparticles.^49^ From the fitted group index data obtained from the Bruggeman model, the effective RI is determined (green line in Fig. 1b). The determined effective RI of the TiO_2_ NP film varies monotonically from n≈1.92 to n≈1.76 for the 400–1,100 nm wavelength spectrum.

### Embossing of TiO_2_ NP-based micro/nanostructured optical coatings

**Figure 2:**
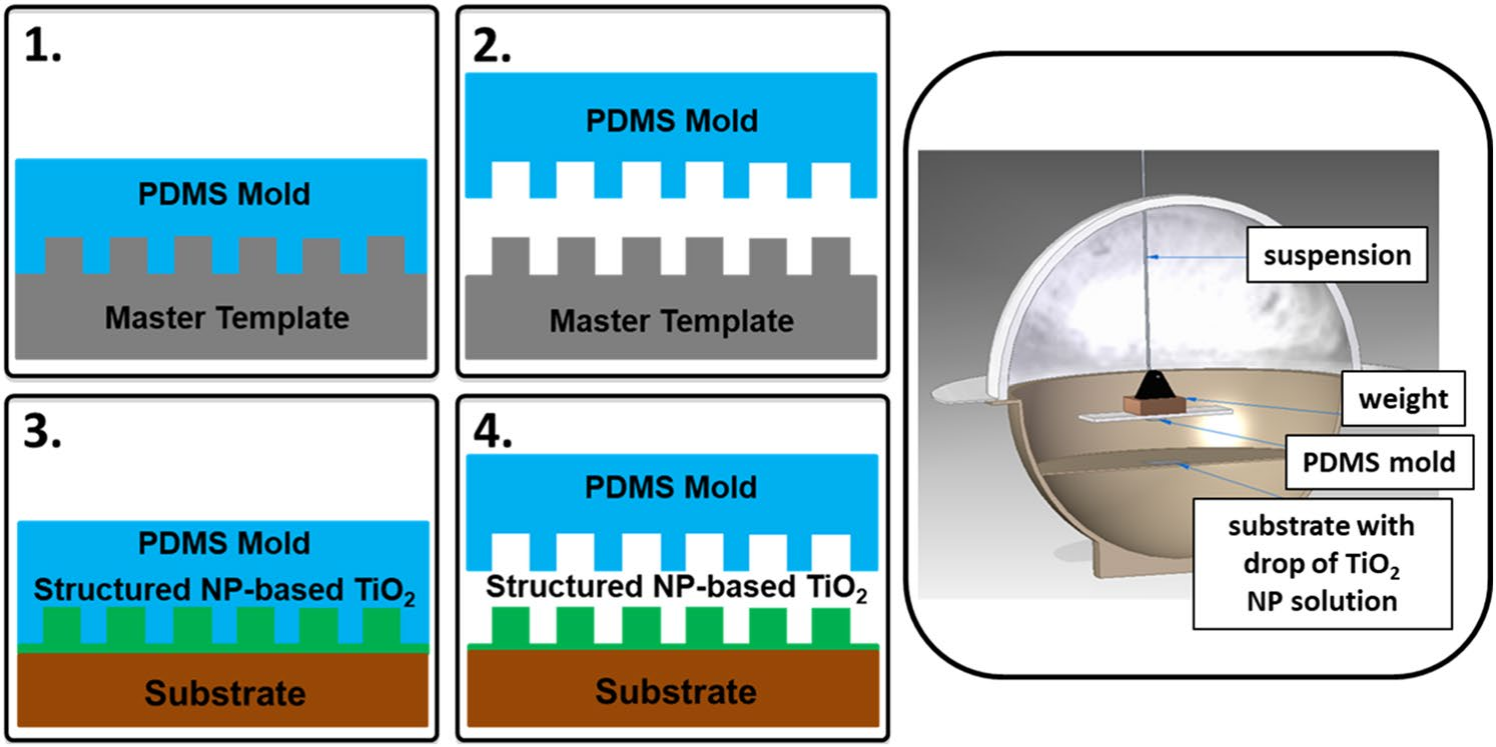
Schematics of the embossing method. Left: Schematic of the process steps involved in the embossing/soft imprinting method. Step 1 shows the making of the PDMS mold from the master template, step 2 the lift-off of the PDMS mold from the master template, step 3 the embossing of the TiO_2_ NP-based structures, and step 4 the lift-off of the PDMS mold from the embossed TiO_2_ NP-based structures. Right: Schematic of the utilized embossing setup.

**Figure 3:**
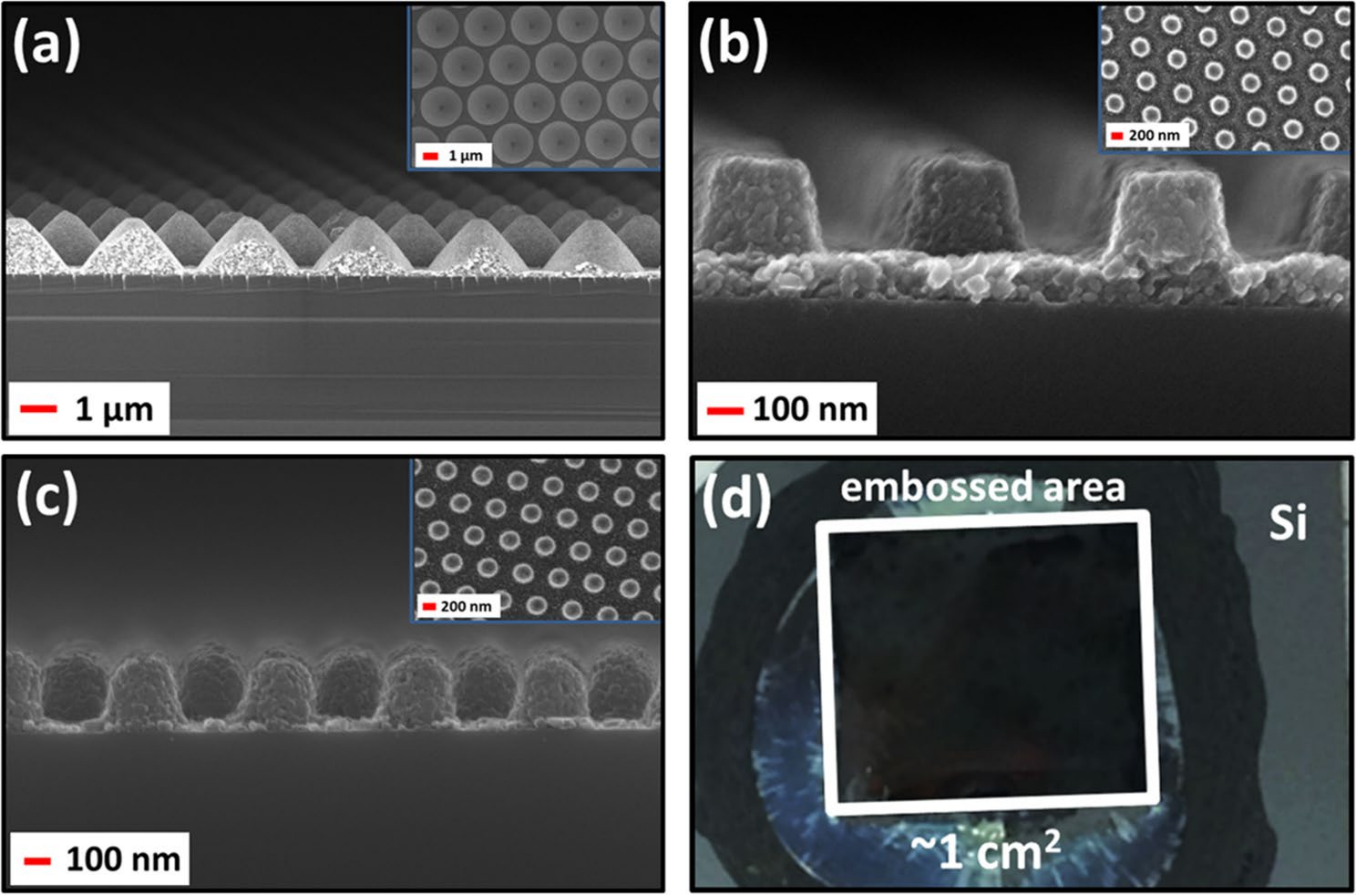
Scanning electron microscopy (SEM) images of the fabricated TiO_2_ NP-based optical coatings on Si. Cross-section SEM images of the embossed (**a**) parabolic microcone array, (**b**) nanodisk-1 array, and (**c**) nanodisk-2 array structures on Si. The top view SEM images are included as insets. (**d**) Camera top view image of the typical embossing area (~ 1 cm^2^).

Figure 2 shows a schematic sketch of the embossing process used to fabricate 3D structures composed of TiO_2_ NPs. Details of the procedures are described under the Methods section. Briefly, using a structured polydimethylsiloxane (PDMS) mold the desired structures are embossed from a TiO_2_ NP solution dispersed on a substrate. Patterns with different micro– and nanostructures were embossed on a variety of substrates such as Si, GaAs, InP, GaN, glass, and ITO coated glass. TiO_2_ NP-based parabolic microcone and nanodisk/pillar arrays are investigated with regard to their anti-reflection properties on Si, GaAs, and InP substrates. The dimensions of the parabolic microcone array structures were based on previous investigations^39^, where these structures were investigated for light extraction enhancement applications in LEDs. Due to their large geometric dimensions, for a major part of the wavelengths considered here such structures are not expected to exhibit Mie scattering. Nevertheless, such microstructures can be developed into gradient refractive index ARCs for solar cells. Such an investigation will require extensive optimization and evaluation which is beyond the scope of the present work. In the present work they are mainly used to show the versatility of the embossing process. Representative SEM images of the micro/nanostructures embossed on Si are shown in Fig. 3. The embossed layer is in intimate contact with the substrate below providing a well-defined optical interface, which is crucial for device applications. On a given sample the array period is uniform over the embossed area, but the heights and diameters of the structures show small variations (< 5%). In all samples, a thin buffer layer (~ 100 nm) remains below the embossed structures. The parabolic microcone array (Fig. 3a) has a hexagonal array period of 3 μm, height of ~ 1.35 μm, and base diameter of ~ 2.6 μm. Two sets of nanodisk/pillar arrays were investigated with similar geometry, for which only the period differs. Nanodisk-1 (Fig. 3b) has a hexagonal array period of 600 nm, height of ~ 240 nm, and top/bottom diameter of ~ 220/300 nm. Nanodisk-2 (Fig. 3c) has a hexagonal array period of 530 nm, height of ~ 260 nm, and top/bottom diameter of ~ 200/300 nm. The camera image in Fig. 3d shows a Si substrate with embossed TiO_2_ NP-based nanodisk arrays; the embossed area (central square ~ 1 cm^2^) is also indicated.

### Anti-reflection properties of the TiO_2_ NP-based optical coatings

**Figure 4:**
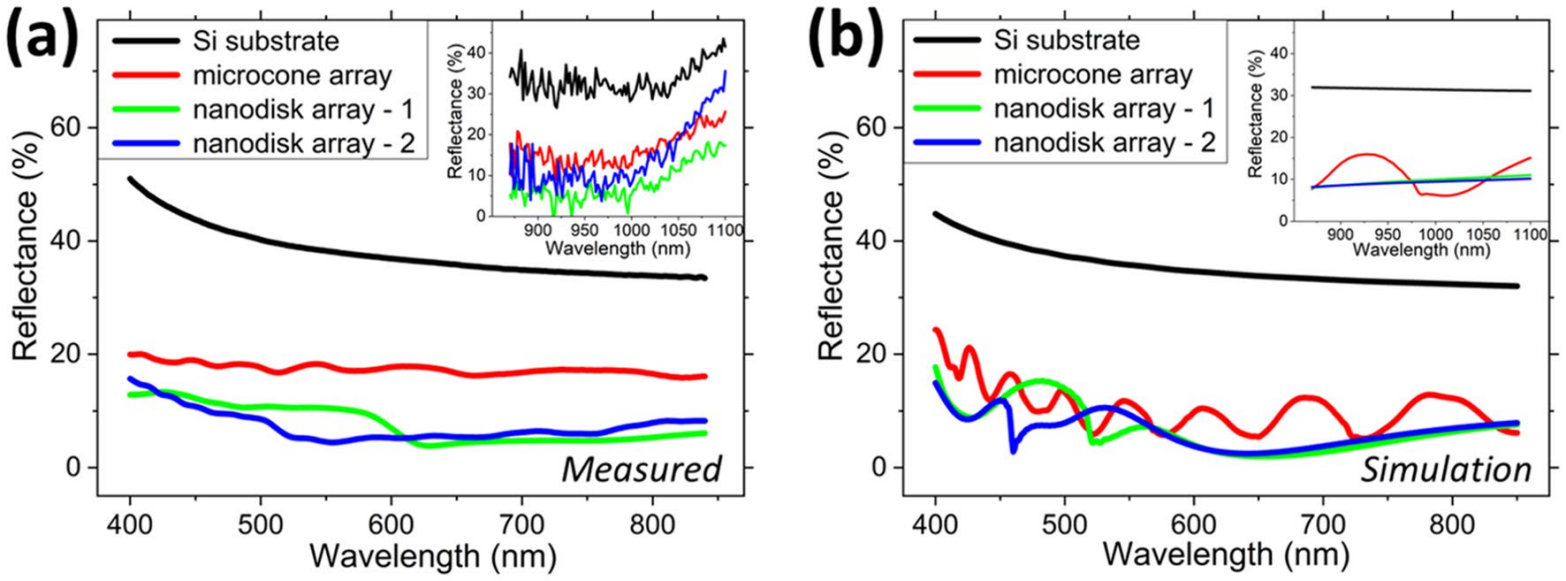
Total reflectance spectra—(**a**) measured and (**b**) simulated—for the embossed TiO_2_ NP-based parabolic microcone, nanodisk-1, and nanodisk-2 arrays on a Si substrate.

The fabricated TiO_2_ NP-based structures are characterized with regard to their anti-reflection properties by spectrally-resolved reflectance (total and diffuse) measurements and finite-difference time-domain (FDTD) simulations. The measured and simulated reflectance spectra for Si substrates with different embossed surface structures are shown in Fig. 4a,b, respectively. The nanodisk array structures show the lowest (average) reflection along the visible-NIR wavelength spectrum. Variations (± 50 nm) of the buffer layer for the embossed structures within the measurement spot size influenced the measured results, whereas for the simulated structures a constant buffer layer thickness (80 nm) was considered. For the fabricated structures, back reflections occur for wavelengths > 1,000 nm (see inset Fig. 4b) which are not absorbed in the Si substrate. This is not considered in the simulated structures. Figure S2 (Supplementary Information) shows the corresponding measured and simulated reflectance spectra for GaAs and InP substrates with the embossed surface structures. The results show similar surface reflectance results for the three substrate materials. This is expected since the RIs of Si, GaAs, and InP for the 400–1,100 nm wavelength spectrum are similar. The measured reflectance is lower than the results reported by Bottein et al.^43^ for a TiO_2_-based optical coating.

The reflectance spectra were simulated using Lumerical’s FDTD simulation tool, using the effective RI of the TiO_2_ NP aggregates (taken from the determined values shown in Fig. 1b (green line)) and the geometric parameters similar to the fabricated structures. The wavelength-dependent optical constants for Si, GaAs, and InP were taken from Palik.^50^ Absorption by the TiO_2_ surface structures was neglected for the simulated wavelength region and the TiO_2_ NP structures were simulated as a ‘homogeneous effective medium’. Scattering effects by the individual TiO_2_ nanoparticles has been neglected in the simulations, which could have an effect for wavelengths < 550 nm (see the measured results in Fig. 1a (dark green line)). A normal incidence plane wave source (TE polarized) and periodic boundary conditions were applied. Polarization effects can be neglected due to the (near) normal incidence angle for both the measurements and simulations, and the symmetry of the structures. The simulations were performed for Si, GaAs, and InP substrates with TiO_2_ NP-based parabolic microcone and nanodisk (nanodisk-1 & nanodisk-2) arrays. For the parabolic microcones, the hexagonal array period is 3 μm, the height is 1.35 μm, and the base diameter is 2.6 μm. For nanodisk-1, the hexagonal array period is 600 nm, the height is 240 nm, and the top/bottom diameter is 220/300 nm. For nanodisk-2, the hexagonal array period is 530 nm, the height is 260 nm, and the top/bottom diameter is 200/300 nm. For all structures, an 80 nm TiO_2_ buffer layer was included. This provided the lowest possible reflectance for a planar thin film with the effective RI value for the TiO_2_ NP layer. Incidentally, this value (80 nm) is close to the average buffer layer thickness (~ 100 nm) in the fabricated samples. Figure S3 of the Supplementary Information shows the influence of the buffer layer thickness on the reflectance and indicates a layer thickness of ~ 80 nm for the lowest (average) reflectance.

An additional lower refractive index conformal layer can be added on the embossed structures functioning as a protection and anti-reflection coating. An example of a SiO_2_ layer coating is shown in Fig. S4 (Supplementary Information), indicating that the structures were not compromised during layer deposition.

### Solar-weighted reflectance of structured TiO_2_ NP-based optical coatings

As a measure for the anti-reflection characteristics of the TiO_2_ optical coatings, a figure of merit (FoM) is determined for the fabricated and simulated structures. Since the total reflectance is the parameter that is directly measured, the FoM is defined based on reflectance. Thus, in context of solar cells, the lower the FoM the better the performance. The reflectance spectra (R(λ)) can be weighed against the solar photon flux for the AM1.5G solar spectrum (ϕ_AM1.5G_(λ)), for the relevant wavelength range for each substrate or solar cell material. For this, a FoM is specified as the solar-weighted reflectivity (Eq. 1).

1 $$ FoM = \frac{{\mathop \smallint \nolimits_{{\lambda_{min} }}^{{\lambda_{bg} }} R\left( \lambda \right) \cdot \phi_{AM1.5G} \left( \lambda \right) \cdot d\lambda }}{{\mathop \smallint \nolimits_{{\lambda_{min} }}^{{\lambda_{bg} }} \phi_{AM1.5G} \left( \lambda \right) \cdot d\lambda }} $$

In this work, for the FoM the minimum wavelength (λ_min_) has been taken as 400 nm, while the maximum wavelength (λ_bg_) related to the bandgap is taken as 1,100, 870, and 920 nm for Si, GaAs, and InP, respectively.

**Figure 5:**
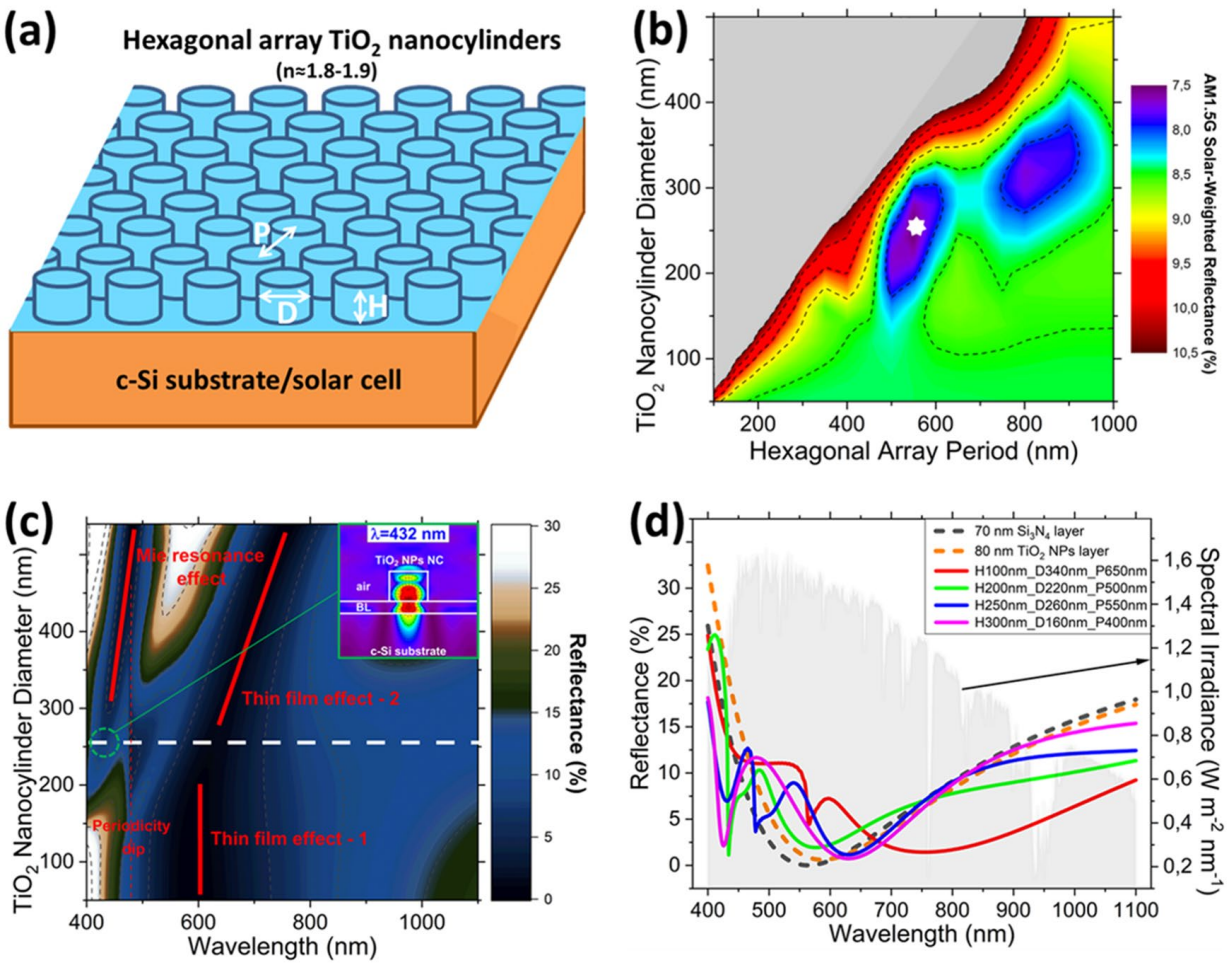
Simulated TiO_2_ (NP-based) nanocylinder (NC) arrays on Si. (**a**) Schematic of the surface structuring. (**b**) Solar-weighted reflectivity (FoM) values for TiO_2_ NP-based NC arrays with a height of 250 nm. The white marker indicates the optimized FoM. (**c**) Contour plot of the reflectance spectra for NC arrays with a height of 250 nm and hexagonal array period of 550 nm. Inset: power field intensity profile at a wavelength of 432 nm. (**d**) Reflectance spectra for the optimized TiO_2_ (NP-based) NC arrays with a height of 100, 200, 250, and 300 nm, respectively. The grey area at the background shows the spectral irradiance for the AM1.5G solar spectrum.

For minimizing the FoM, a simulation study was performed to investigate the most optimal geometrical parameters for TiO_2_ NP-based nanocylinder (NC) array structures. A plane wave source (TE polarized) at normal incidence was used in combination with periodic boundary conditions. All above bandgap light transmitted into the semiconductor is assumed to be absorbed. The RI data for the TiO_2_ NP-based structures is taken from Fig. 1b and any absorption is neglected for the simulated wavelength spectrum. A schematic sketch of the simulated structures is shown in Fig. 5a. Four NC heights (H = 100, 200, 250, and 300 nm) were investigated. For each case, the NC diameter (D) was swept from 50–500 nm with 20 nm steps and the (hexagonal) array period (P) was swept from 100–1,000 nm with 50 nm steps. An optimized buffer layer thickness of 80 nm was used for all structures. The FoM data for the heights of 100, 200, and 300 nm are included in Fig. S5 of the Supplementary Information. Figure 5b shows the FoM data obtained for NC arrays with a height of 250 nm, similar to the height of the fabricated nanodisk array structures. Two minima can be identified (purple areas in Fig. 5b). A period of 550 nm and a diameter of 260 nm results in the lowest FoM (~ 7.5%) and is indicated by the white marker in Fig. 5b.

Figure 5c shows the reflectance spectra for NCs with a period of 550 nm and a height of 250 nm, where the NC diameter is plotted versus the wavelength of the incident light. Different effects leading to the lowering of the surface reflection can be identified. One effect is due to the buffer layer (*thin film effect – 1*) that occurs for the NC diameter range of ~ 50–250 nm. A second effect occurs for a NC diameter range of ~ 200–550 nm due to an effective medium effect (*thin film effect – 2*). The NC-air medium acts as a thin film for which the effective index can be obtained using Eq. 2. This effective index is a volume-weighted average of the RIs of the NP-based TiO_2_ NCs (n_NPsTiO2_) and air (n_air_). The thickness of this effective thin film layer is determined by the height of the NCs. Thus, in principle, for wavelengths longer than the period of the NCs, this type of structuring can be seen as a dual layer ARC; where the RI of the top layer can be tuned by optimizing the diameter and periodic spacing of the NC arrays.

2 $$ n_{eff} = n_{{NPsTiO_{2} }} \cdot \left( {\frac{{\pi \cdot d^{2} }}{{3 \cdot \sqrt 3 \cdot p^{2} }}} \right) + n_{air} \cdot \left( {1 - \frac{{\pi \cdot d^{2} }}{{3 \cdot \sqrt 3 \cdot p^{2} }}} \right) $$

This thin film effect was confirmed by simulations when sweeping the height from 100–1,000 nm by using both the effective medium layer and the NC array structuring. The TiO_2_ NP-based NC arrays with a hexagonal array period of 550 nm and diameter of 260 nm, results in an effective index of n_eff_≈1.12. A third effect that occurs is due to Mie resonances, which is observed in the wavelength region of ~ 400–450 nm, for a NC diameter range of ~ 200–550 nm. The reflectance spectrum for TiO_2_ NCs with a height of 250 nm, diameter of 260 nm, and hexagonal array period of 550 nm is indicated by the white dashed horizontal line in Fig. 5c. The inset in Fig. 5c shows the power field intensity profile at a wavelength of ~ 432 nm. The occurring Mie resonance results in forward scattering into the substrate.^4-9,40-44^ In hexagonal arrays, reflectance dips due to a grating effect (Wood-Rayleigh anomaly) occur at ‘p/2′ and ‘(p/2)·(3)^1/2^′, where ‘p’ is the period. In the investigated wavelength range, for the hexagonal period spacing of ~ 550 nm the dip appears at a wavelength of ~ 480 nm.

Figure 5d shows the reflectance spectra (simulation) for the optimal NC diameter and hexagonal array period for different NC heights. Additionally, reference reflectance spectra are included for a 70 nm Si_3_N_4_ layer on Si, related to the lowest reflectivity for a single thin film ARC, and the optimized 80 nm layer consisting of the mixture of anatase and rutile TiO_2_ NPs on Si. The simulated reflectance for the array period of 550 nm, disk height of 250 nm, and disk diameter of 260 nm (horizontal line in Fig. 5c) is also included in Fig. 5d. In the investigated wavelength range, for the different array periods (400, 500, 550, and 650 nm) the associated reflectance dips due to Wood-Rayleigh anomaly appear at ~ 432 nm (p = 500 nm), ~ 480 nm (p = 550 nm), ~ 562 nm (p = 650 nm). As discussed earlier, different wavelength-dependent optical effects are present and the associated reflectance characteristics also vary with the structure geometry (period and disk dimensions). For example, following the data shown for the specific structure in Fig. 5c the reflectance dips at ~ 432 and ~ 480 nm correspond to a Mie resonance and Wood-Rayleigh anomaly, respectively, while the broad dip around 625 nm is due to a thin film effect. Here, our focus is on the FoM obtained with the different optimized structures.

The FoMs for the different types of fabricated and simulated TiO_2_ structures were determined by using Eq. 1. Examples of the FoMs for the simulated structures on Si are included in Fig. S6 of the Supplementary Information. The data for the nanodisk arrays refer to the optimized disk dimensions for the given periods. For array periods of 400, 500, and 550, the FoM values were between 7–8% in the simulation wavelength spectrum of 400–1,100 nm. The structures with a period of 650 nm, diameter of 340 nm, and height of 100 nm showed the lowest FoM (~ 6.6%). With respect to the fabricated structure geometries, the lowest FoM values for the parabolic microcone and nanodisk arrays on Si were found to be ~ 10% and ~ 7%, respectively. The geometry and spacing of nanodisk-1 and nanodisk-2 are comparable to the optimal NC array structures for a NC height of 250 nm (see Fig. 5b). The average diameter of nanodisk-1/nanodisk-2 is close to the optimal diameter (260 nm) for NCs with a height of 250 nm and hexagonal array period of 550 nm. Little influence on the FoM value was observed due to their slightly different periods.

Mie resonator structures can have omnidirectional anti-reflection properties and have advantages compared to a simple layer coating, especially for higher off-normal incidence angles of the incident light.^4-9^ Additionally, these type of Mie resonator structures typically show scattering cross-sections larger than their geometrical cross-section. Scattering from such structures can also result in longer optical path lengths in the absorbing medium, providing better absorption of near bandgap light wavelengths in thin film solar cell geometries. Representative simulation data for the absorbed power distribution (for incident light with a wavelength of 650 nm) and solar-weighted absorbance along a 10 µm vertical penetration depth into Si, is included in Fig. S7 of the Supplementary Information. The results show that a slightly higher solar-weighted absorbance (~ 80%) is obtained for the mixture of anatase and rutile TiO_2_ NP-based nanodisk arrays compared to an optimized single layer consisting of the mixture of anatase and rutile TiO_2_ NPs (~ 78%) within this first 10 µm layer of the Si substrate (see Fig. S7).

The FoM values obtained for the TiO_2_ nanodisk structures in this work (~ 7%) are comparable to the ones (~ 5.9%) obtained with Si NC arrays directly structured on Si, when considering the same wavelength spectrum for the FoM.^3^ Here we note that with additional conformal Si_3_N_4_ coating on Si NC arrays^3^ or using multilayer dielectric coatings,^21,24,27^ broadband surface reflectance can be reduced to less than 1.5–3%. However, these approaches require either direct etching of the solar cell material and/or multiple process steps to fabricate the ARC coating. Preliminary simulations assuming rutile TiO_2_ NP-based nanodisk arrays, with a similar fill factor (0.57), geometry, and spacing as is reported for nanodisk-1, indicates that surface reflections can be reduced to ~ 4–5% (see Fig. S8 of the Supplementary Information). Simulation data for the absorbed power distribution (for incident light with a wavelength of 650 nm) and solar-weighted absorbance along a 10 µm vertical penetration depth into Si for this type of structuring, is included in Fig. S7. This data indicates that an even higher solar-weighted absorbance (~ 85%) can be obtained within the 10 µm top portion of the Si substrate/solar cell due to better anti-reflection and scattering properties. These results indicate that adding TiO_2_ NP-based nanodisk array structures provide broadband anti-reflection, while avoiding direct structuring of the solar cell material.

### Embossed TiO_2_ NP-based nanodisk arrays on solar cells

**Figure 6:**
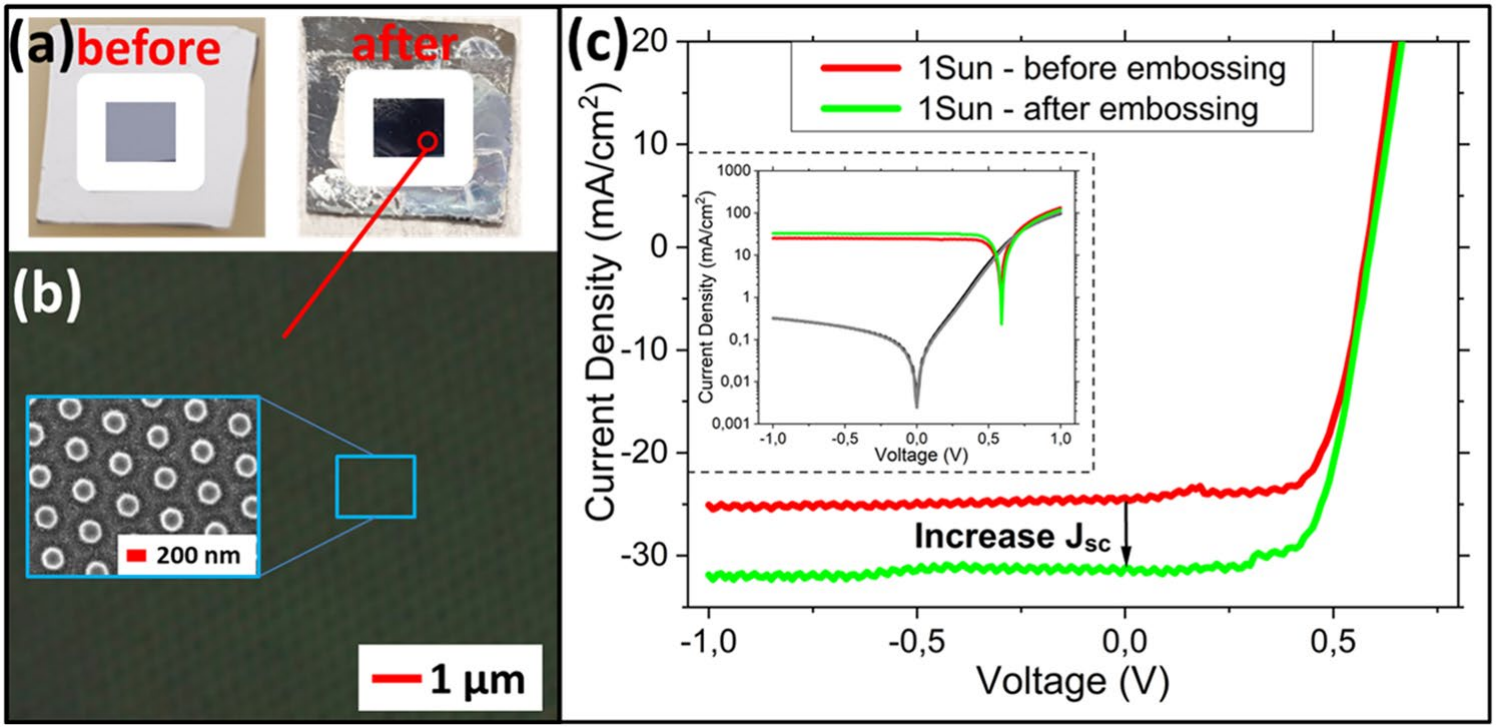
Embossing result and solar cell I-V characteristics for TiO_2_ NP-based nanodisk arrays embossed on a prefabricated planar single-junction Si solar cell. (**a**) Camera top view image of the Si solar cell surface before and after embossing (indicated within the white frame). (**b**) Microscope and SEM top view image of the TiO_2_ NP-based nanodisk arrays embossed on the Si solar cell. (**c**) The I-V characteristics of the Si solar cell before and after embossing. Inset: logarithmic scale of the I–V characteristics, including the dark currents (black and grey lines).

As a proof of principle, TiO_2_ NP-based nanodisk array structures were embossed on prefabricated planar single-junction Si, GaAs, and InP solar cells (typical size of 0.5 × 0.5 cm). The embossed nanodisk structures are similar to those shown in Fig. 3b. The embossing results of the nanodisk arrays on the Si solar cell is shown in Fig. 6a and a representative top view optical microscope and SEM image are shown in Fig. 6b. The solar cells were not optimized for their efficiency. Here the focus is only on demonstrating the beneficial effect of the embossed anti-reflection TiO_2_ nanodisk array optical coating. Thus, only the short-circuit current density (J_sc_) characteristics before and after embossing are compared. However, a slight increase (~ 10–20 mV) of the open-circuit voltage (V_OC_) was observed for the embossed solar cells.

Reflectance spectra were taken before and after embossing on the solar cells; this area excludes the metal contact pads. As expected, the spectra are similar to the ones shown in Figs. 4a and S2 (Supplementary Information). The (embossed) solar cells were characterized by I-V measurements. A simple solar lamp (AM1.5; 1 Sun) was used for illumination. The results for the Si solar cell before and after embossing are included in Fig. 6c. The results for the GaAs and InP solar cells are given in the Supplementary Information in Fig. S9. For all three solar cell materials an appreciable increase of the J_sc_ is observed after embossing. The dark current was unaltered after embossing, indicating that the solar cell device was not compromised due to the embossing process. For the Si solar cell a value of J_sc,before_≈24.36 mA/cm^2^ and J_sc,after_≈31.18 mA/cm^2^ was measured before and after embossing, respectively. This resulted in an increase of ~ 6.81 mA/cm^2^, which is equivalent to an increasing factor of ~ 1.3 times. For the GaAs and InP solar cells values of J_sc,before_≈8.31 mA/cm^2^ and J_sc,before_≈14.86 mA/cm^2^ were measured before embossing and after embossing these values were J_sc,after_≈10.54 mA/cm^2^ and J_sc,after_≈18.85 mA/cm^2^, respectively. This is equivalent to an increase of ~ 2.23 and ~ 3.99 mA/cm^2^ for the GaAs and InP solar cells, respectively, resulting in an increasing factor of ~ 1.3 times for both types of solar cells. The obtained J_sc_ enhancement agrees well with data reported in a simulation study for a similar type of structuring on InP solar cells.^44^.

## Conclusion

In this work (a mixture of anatase and rutile) titanium dioxide (TiO_2_) nanoparticle (NP)-based optical coatings are investigated for broadband omnidirectional anti-reflection applications. NP assemblies are interesting for light manipulation functions since their effective refractive index (RI) can be tuned by engineering the fill factor, particle size, and the RI of the individual NPs. A table-top, cost-effective, low pressure (~ 0.1 bar), and low temperature (< 100 °C) embossing method was developed and used to emboss micro- and nanostructured NP-based optical coatings on various substrate surfaces. Due to the low temperature processing, these optical coatings can be applied in a broader scope of material combinations.

A NP-based layer assembly was used to investigate the optical properties for the 400–1,100 nm wavelength spectrum. The results show that this layer is non-absorbing for wavelengths > 400 nm and the NPs show scattering effects for wavelengths < 550 nm. A fill factor of ~ 0.57 was determined for the TiO_2_ NP-based thin films by using a combination of Fabry-Pérot oscillations and a Bruggeman model, resulting in an effective RI of ~ 1.92–1.76 for the 400–1,100 nm wavelength spectrum.

TiO_2_ NP-based parabolic microcone and nanodisk arrays were embossed on Si, GaAs, and InP substrates. The embossed structures were characterized by spectrally-resolved total, specular, and diffuse reflectance measurements and by finite-difference time-domain (FDTD) simulations for the wavelength spectrum of 400–1,100 nm. A figure of merit (FoM) for anti-reflection is defined as the solar-weighted reflectivity. FoM values as low as ~ 7% were determined for the mixture of anatase and rutile TiO_2_ NP-based nanodisk array structures. FDTD simulations for optimizing the geometry and spacing of TiO_2_ nanocylinder (NC) array structures based on the mixture of anatase and rutile NPs, show a possible FoM of ~ 6.6%.

Finally, as a proof of principle, TiO_2_ NP-based nanodisk arrays were embossed on prefabricated planar single-junction Si, GaAs, and InP solar cells. After embossing the TiO_2_ nanodisk arrays, all three solar cell materials show strong reduction in surface reflection and an appreciable increase (~ 30%) in the short-circuit current densities (J_sc_).

## Methods

### Colloidal TiO_2_ nanoparticles

For the TiO_2_ nanoparticles (NPs), a water-based colloidal solution (33–37 wt%; Sigma Aldrich) of a mixture of anatase and rutile TiO_2_ NPs was used; the average size is ~ 50 nm, however larger particles, up to 150 nm, could be present (see Fig. S1 in the Supplementary Information). These particle size distributions allow fabrication of microstructures and nanostructures (of a few hundred nm in dimensions). However, it can also result in a buffer layer (50 to 150 nm thick). To fabricate smaller structures and to reduce the buffer layer thickness, much smaller (10–20 nm) and uniform nanoparticle sizes are required. TiO_2_ NP layers on Si and on glass were obtained by spin coating at a spin-off speed of 1,000 rpm, by using the original TiO_2_ NPs solution concentration. The typical obtained layer thickness was ~ 1.7–1.9 µm. High-resolution X-ray diffraction (HR-XRD) data for a spin coated (mixture of anatase and rutile) TiO_2_ NP-based film on a glass substrate (~ 1–9 μm thickness) has been included in Fig. S10 of the Supplementary Information. The HR-XRD data indicates both the presence of the anatase and rutile crystalline phase.

### Embossing of micro- and nanostructured TiO_2_ NP-based optical coatings

Three different master templates were used to create the PDMS stamps for fabricating the TiO_2_ NP-based parabolic microcone, nanodisk-1, and nanodisk-2 arrays. For the parabolic microcones, a parabolic microcone master template was used with a hexagonal array period of 3 μm, height of ~ 1.7 μm, and base width of ~ 2.8 μm. For the nanodisk-1 structure a nanopillar-shaped master template was used with a hexagonal array period of 600 nm, average diameter of ~ 300 nm, and height of ~ 500 nm. For the nanodisk-2 structure, a dome-shaped master template was used with a hexagonal array period of 530 nm, average diameter of ~ 300 nm, and height of ~ 450 nm. The PDMS molds were fabricated by pouring the PDMS precursors (Sylgard 184 Silicone Elastomer; two parts with 10:1 mix ratio) on the master template, degassing it in a desiccator for 30 min, heating it at 100 °C for 50 min and finally lifting off the molded PDMS film. Thus, an inverted replica of the pattern of the master template is obtained in the PDMS stamp. A typical PDMS area size of ~ 1 cm^2^ was used in this work. However, the embossing area can in principle be scaled-up by modifying the embossing setup. A drop of the TiO_2_ NPs solution (~ 20 μL) was first dispersed on the substrate surface and manually spread using the pipette. Then, the PDMS mold was pressed down with a controlled pressure (~ 100 g/cm^2^ = 0.1 bar) and retained until the NPs solution was dried out. During this process, the NPs infill the open regions in the PDMS mold, forming the 3D patterns. Here we note that in the process of pressing the PDMS mold, the excess solution squeezes out at the open sides of the PDMS mold. To speed up the evaporation of the solvent, the samples were heated (to 100 °C) for 15 min. Finally, the PDMS stamp is peeled-off in order to obtain the patterns with the compacted TiO_2_ NPs on the substrate surface. The optimal settings for pattern formation were obtained for a NP concentration of ~ 35 wt% and applied pressure of ~ 100 g/cm^2^. During the embossing process, the desiccator is evacuated to prevent the formation of air bubbles. The geometry of the embossed structures was smaller than the geometry of the master template. This is expected since the infilling of the PDMS stamp occurs in solution form and after solvent evaporation only TiO_2_ NPs are left behind. The embossed microstructures showed a smaller height and base width/diameter due to the drying process of the embossed TiO_2_ NP-based assemblies, where the same hexagonal array period was maintained. The embossed nanostructures predominantly showed a smaller height than the master templates, with a smaller average diameter, and where the same hexagonal array period was maintained.

### Spectrophotometry

The total and diffuse reflectance and transmittance spectra were measured using a UV/Vis/NIR spectrophotometer (Perkin Elmer; Lambda 950) equipped with an integrating sphere. The beam spot size was typically ~ 1.5 mm. The specular reflectance was determined using the total and diffuse reflectance. The absorbance (A) was determined by A(%) = 100%-R(%)-T(%); where R(%) is the total reflectance and T(%) the total transmittance.

### I-V characterization

The I-V characterization of the Si, GaAs, and InP solar cells were measured using a home-built probe setup, an Agilent B1500A Semiconductor Device Analyzer, and a solar lamp (100 mW/cm^2^) simulating the AM1.5G illumination (L.O.T.-Oriel).

### Simulations

For the optical simulations Lumerical’s finite-difference time-domain (FDTD) Solutions simulation tool was used.

## Supplementary information

Supplementary file1 (PDF 2241 kb)
