# Supplementary material for: Embossed Mie resonator arrays composed of compacted TiO2 nanoparticles for broadband anti-reflection in solar cells
Source: Sci Rep. 2020 Jul 27;10:12527. doi: 10.1038/s41598-020-69518-6 (PMC7385151; doi:10.1038/s41598-020-69518-6)
Supplement: Supplementary file 1 — Supplementary file1 (PDF 2241 kb) [file 41598_2020_69518_MOESM1_ESM.pdf]

# Supplementary Information

## Embossed Mie resonator arrays composed of compacted $TiO_2$ nanoparticles for broadband anti-reflection in solar cells

*Dennis Visser, Ding Yuan Chen, Yohan Désières, Ajith Padyana Ravishankar, and Srinivasan Anand*

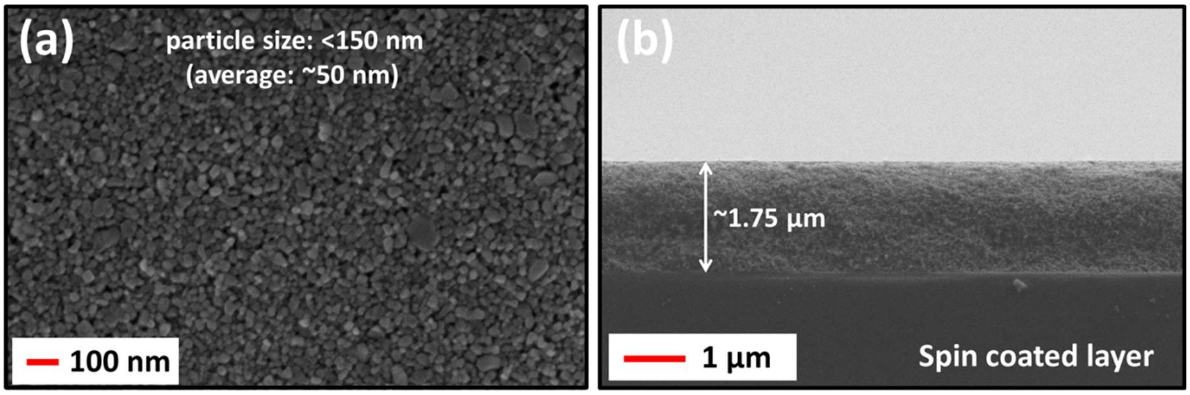


**Figure S1.** Scanning electron microscopy (SEM) images of (a) a top view of the NP size distribution and (b) a spin coated $TiO_2$ NP-based layer with a thickness of ~1.75 µm on a Si substrate.

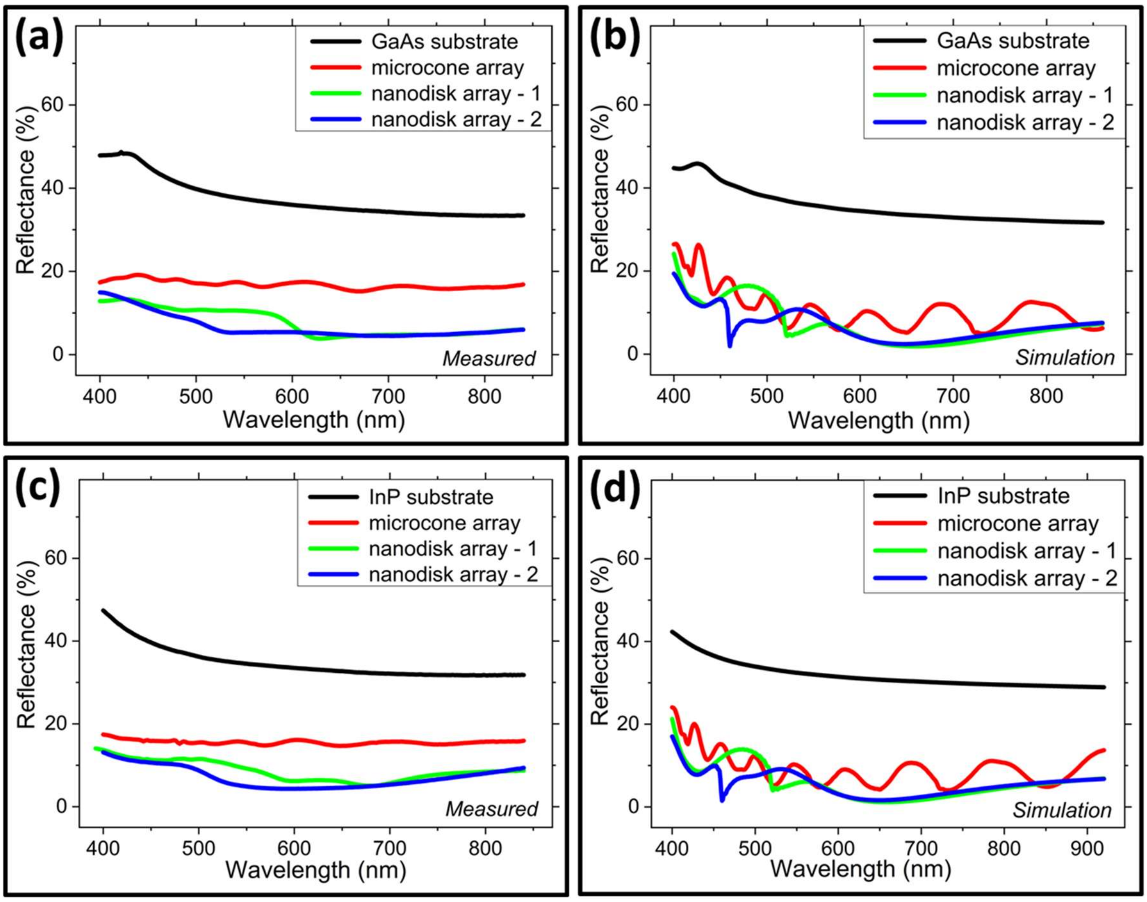


**Figure S2.** Measured and simulated total reflectance for the embossed $TiO_2$ NP-based parabolic microcone, nanodisk-1, and nanodisk-2 arrays on a GaAs ((a) and (b)) and InP ((c) and (d)) substrate.

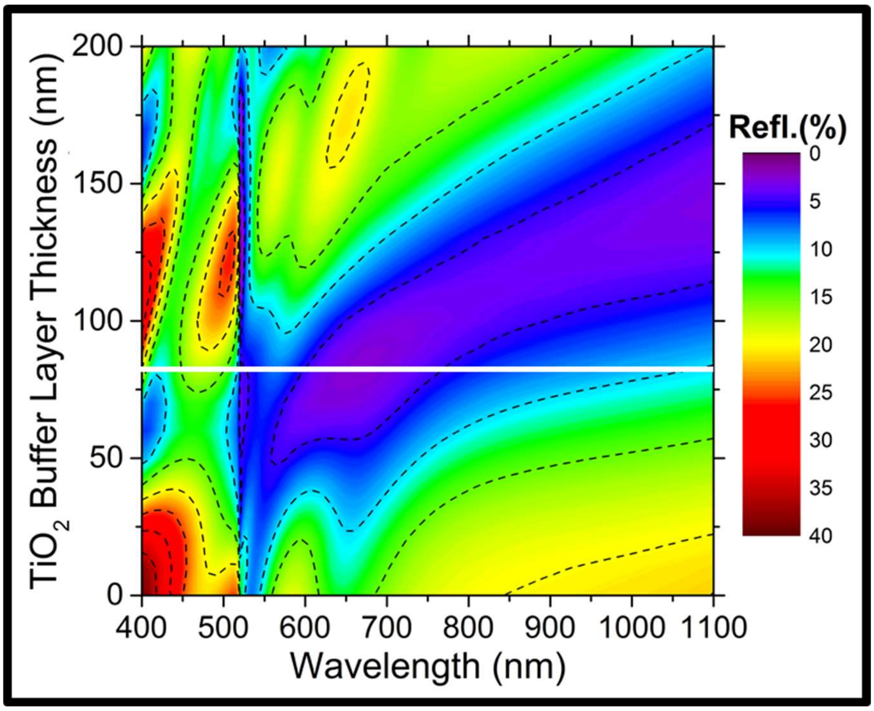


**Figure S3.** FDTD simulations showing the influence of the buffer layer thickness (thickness of 0-200 nm) below the nanodisk-1 array structures. The white horizontal line depicts the optimal buffer layer thickness (80 nm).

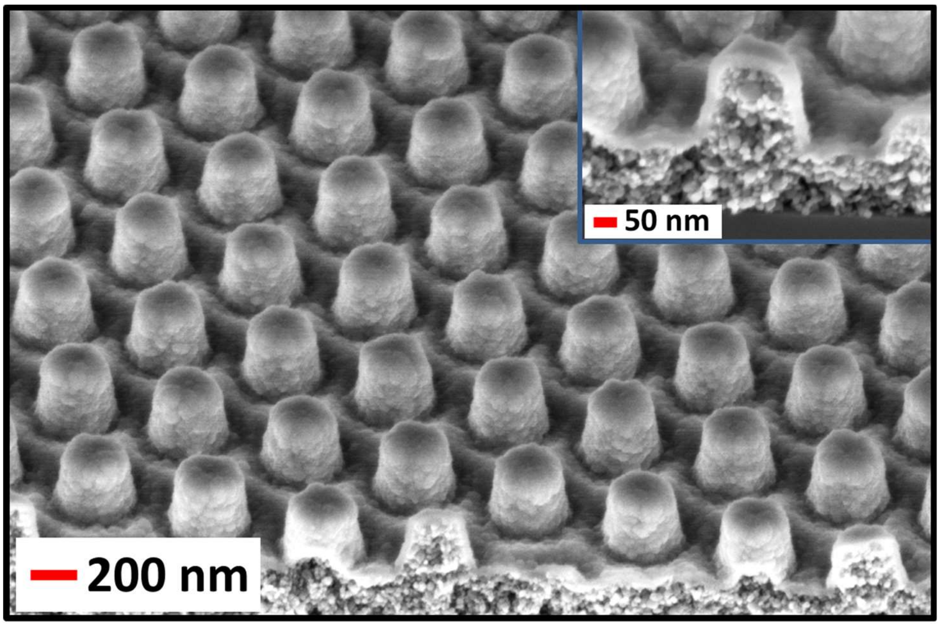


**Figure S4.** Scanning electron microscopy (SEM) images of a $SiO_2$ coating/protection layer on the $TiO_2$ NP-based nanodisk-1 array structures. Inset: zoom-in cross-section SEM image.

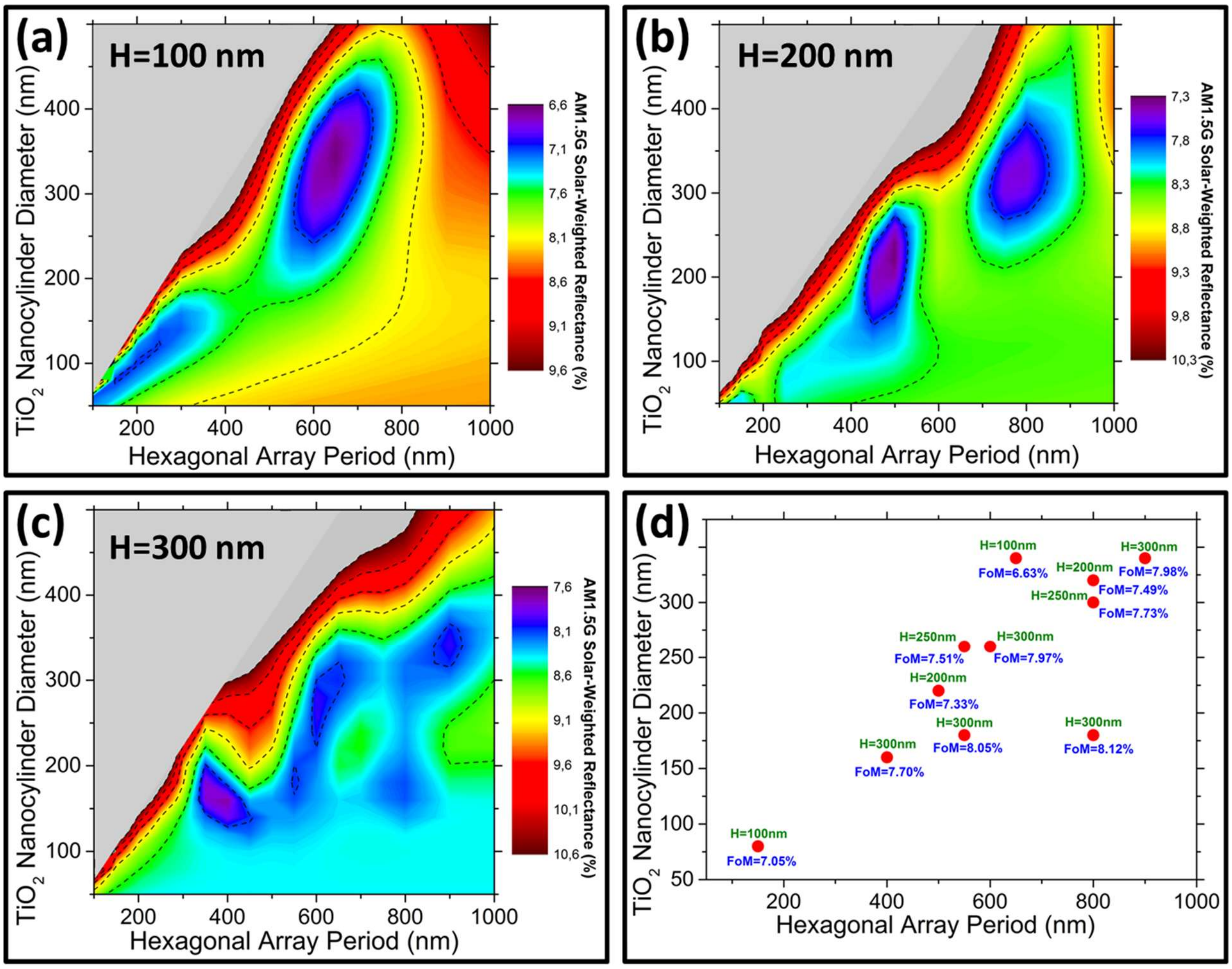


**Figure S5.** FDTD simulations regarding the figure of merit (FoM) data for nanocylinders (NCs) with a height of (a) 100, (b) 200, and (c) 300 nm. The $TiO_2$ NC diameter (50-500 nm) and hexagonal array period (100-1000 nm) were swept. (d) Shows the lowest FoM values for the data obtained in (a)-(c); where the NC height (green) and FoM (blue) values are indicated for each data point (red).

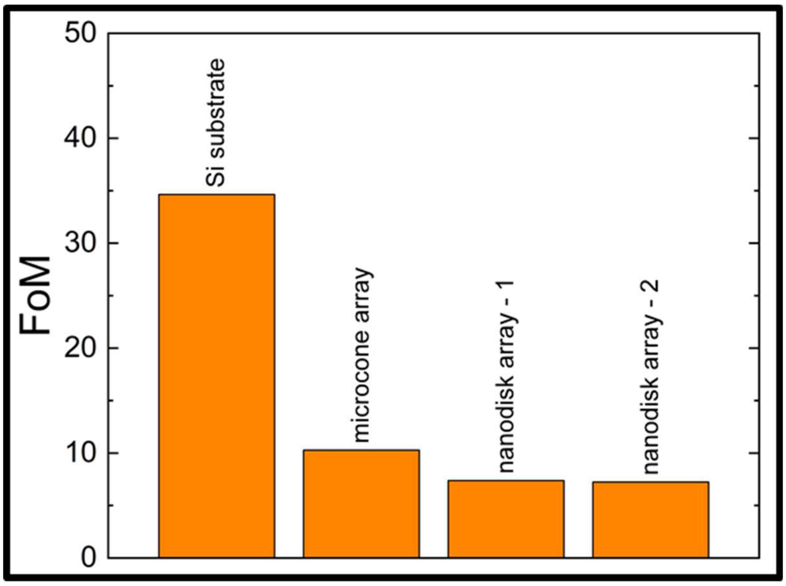


**Figure S6.** FoM values for the simulated (NP-based) $TiO_2$ structures on a Si substrate.

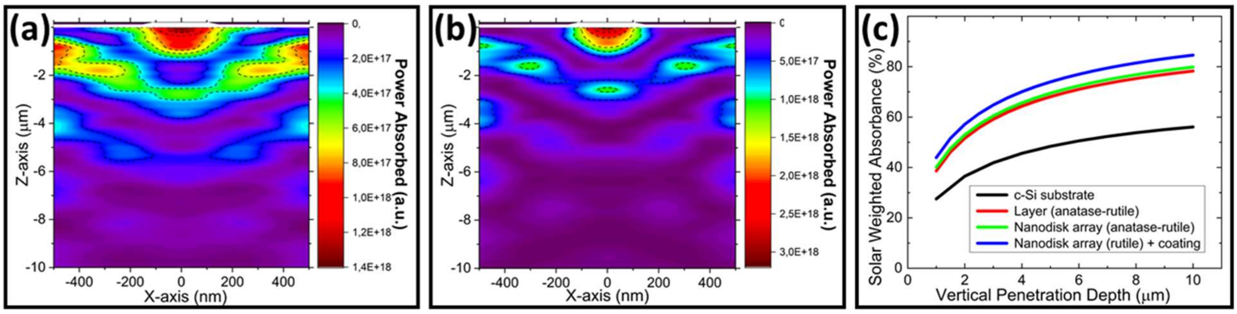


**Figure S7.** (a) and (b) Simulated absorbed power field distributions along the top 10 µm portion of a Si substrate for the mixture of anatase and rutile, and rutile (with $SiO_2$ layer coating) $TiO_2$ NP-based nanodisk array structures on Si; for an incident wavelength of 650 nm. (c) Solar-weighted absorbance for a vertical penetration depth varying from 1-10 µm, shown for the Si substrate, optimized layer based on a mixture of anatase and rutile $TiO_2$ NPs, nanodisk-1 array based on a mixture of anatase and rutile $TiO_2$ NPs, and rutile $TiO_2$ nanodisk-1 array with optimized $SiO_2$ protection/anti-reflection layer coating.

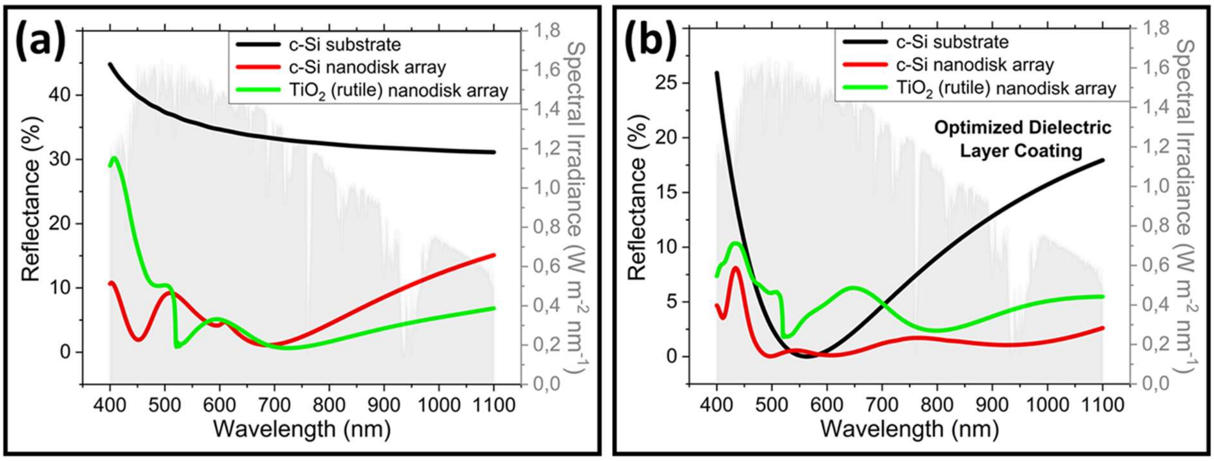


**Figure S8.** The simulated reflectance spectra for rutile $TiO_2$ NP-based (fill factor: 0.57) nanodisk-1 arrays on Si versus the direct structuring of Si based on optimized nanodisk arrays. The reflectance spectra are shown in (a) without additional dielectric coating and in (b) with optimized dielectric coating. As dielectric coating layer, $Si_3N_4$ and $SiO_2$ were used for the Si nanodisk arrays and the rutile $TiO_2$ NP-based nanodisk-1 arrays, respectively.

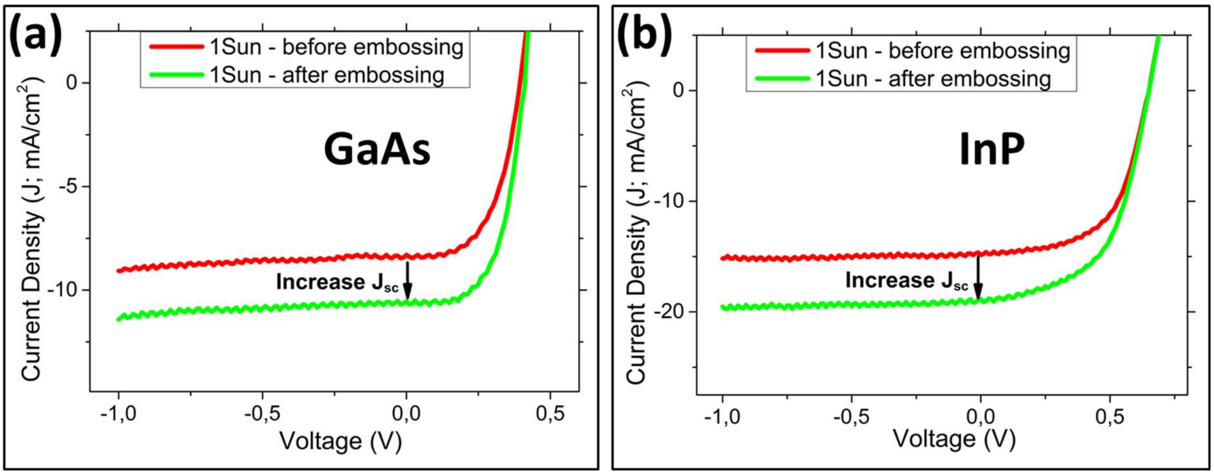


**Figure S9.** The I-V characteristics of the (a) GaAs and (b) InP solar cells before and after embossing.

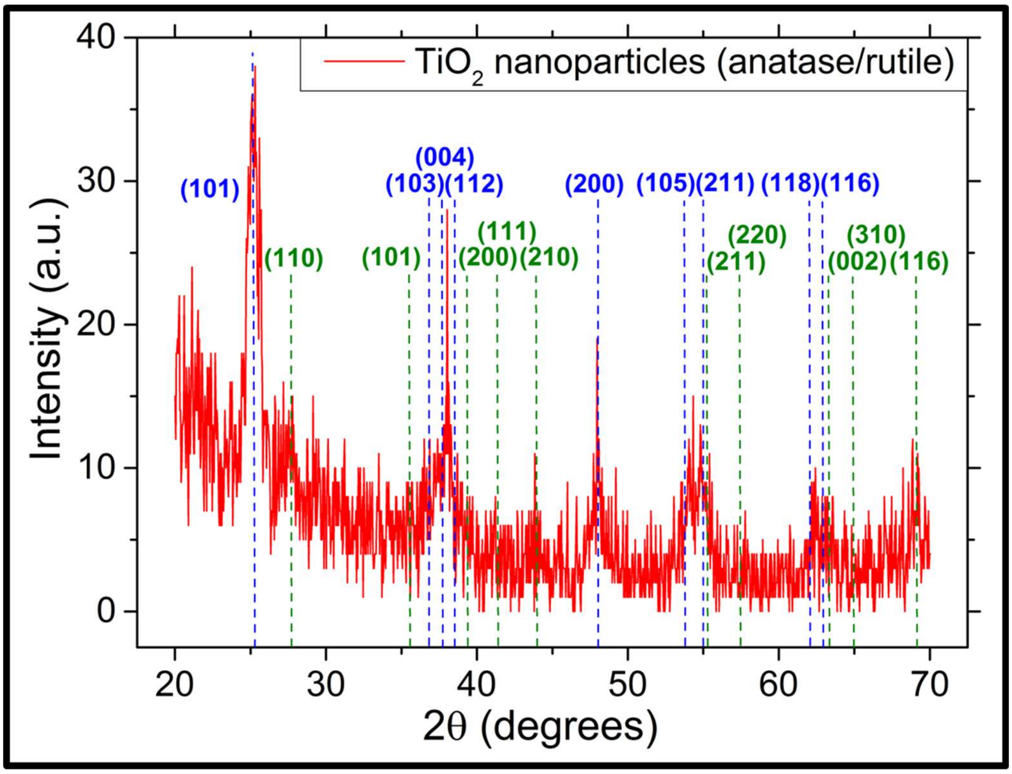


**Figure S10.** High-resolution X-ray diffraction (HR-XRD) data for the mixture of anatase and rutile $TiO_2$ NP-based film (~1.9 µm) spin coated on a glass substrate. The HR-XRD data indicates both the presence of the anatase (blue) and rutile (green) crystalline phase.[1,2]


[1] Thamaphat, K., Limsuwan, P. & Ngotawornchai, B. Phase characterization of $TiO_2$ powder by XRD and TEM. *Kasetsart J. Nat. Sci.* **42**, 357-361 (2008).

[2] Theivasanthi, T. & Alagar, M. Titanium dioxide ($TiO_2$) nanoparticles XRD analyses: an insight. *arXiv:1307.1091* *[physics.chem-ph]* (2013).
